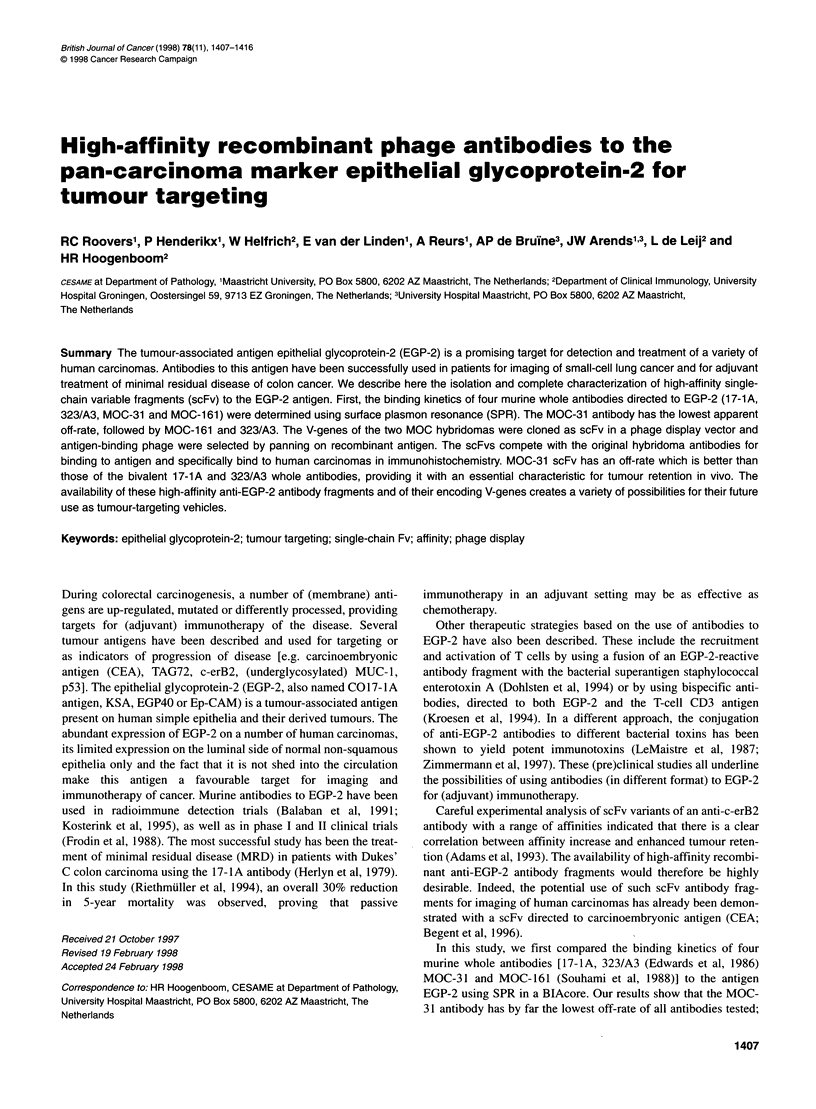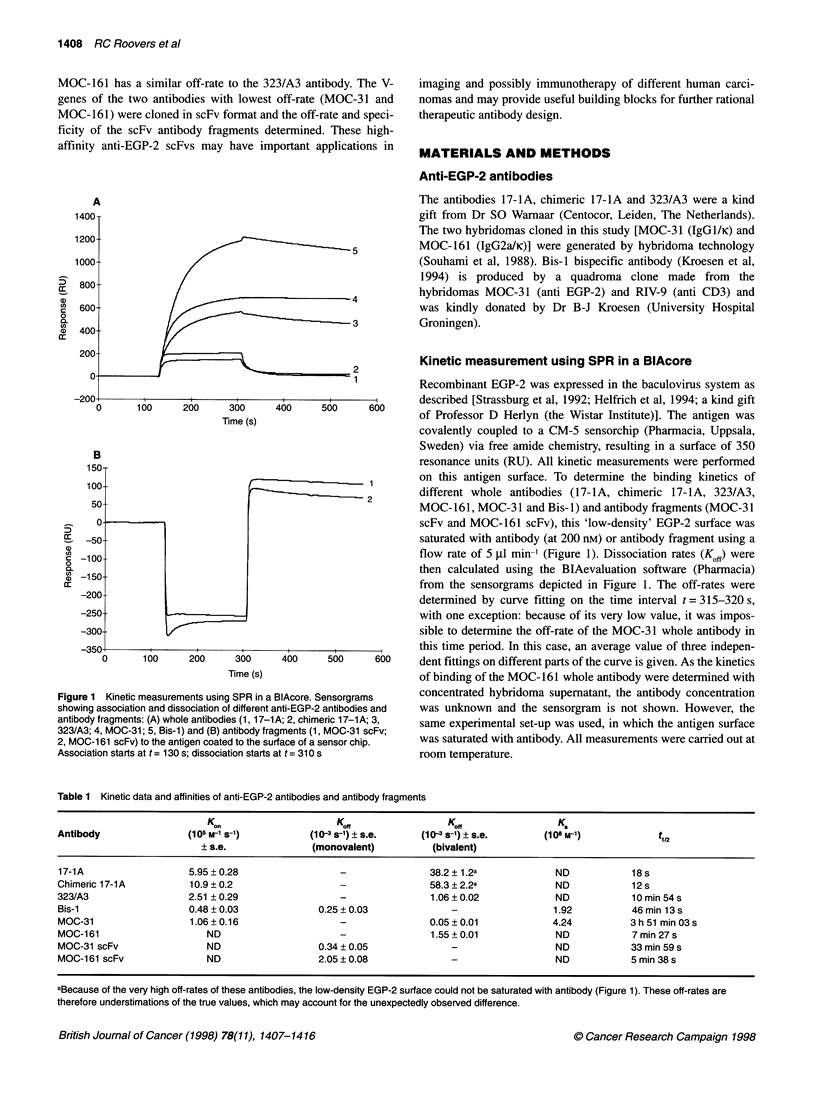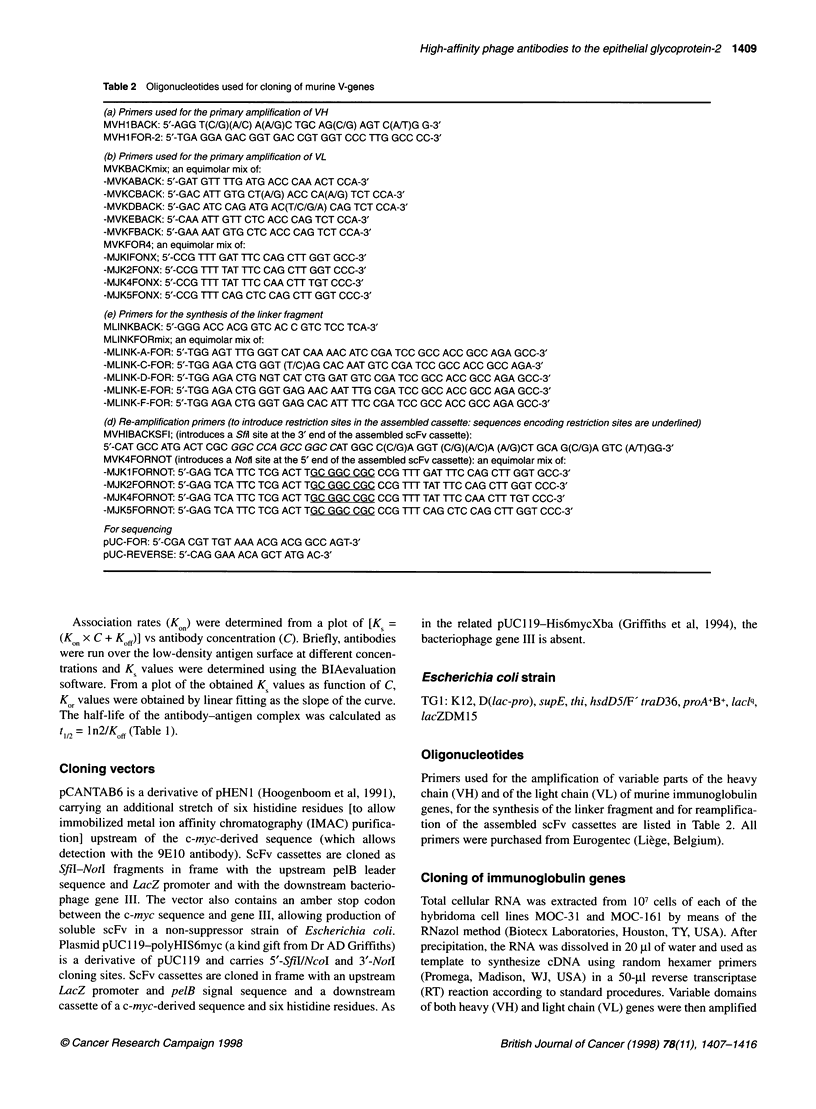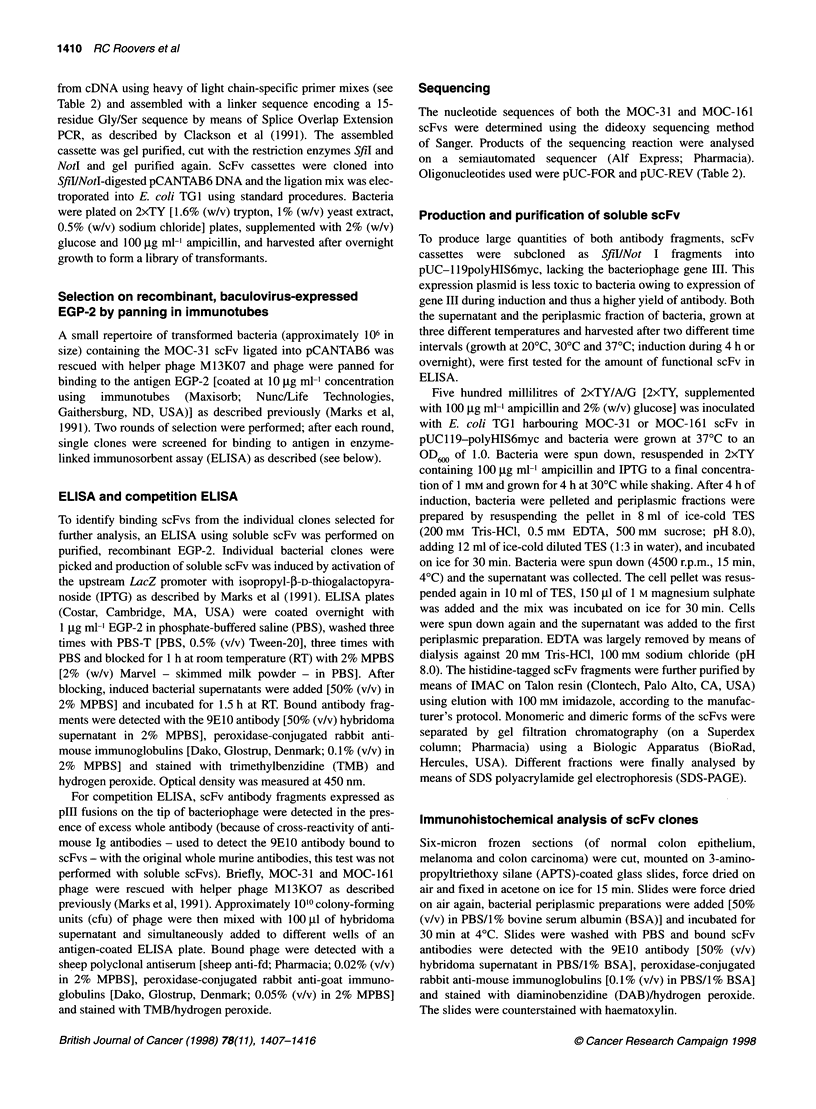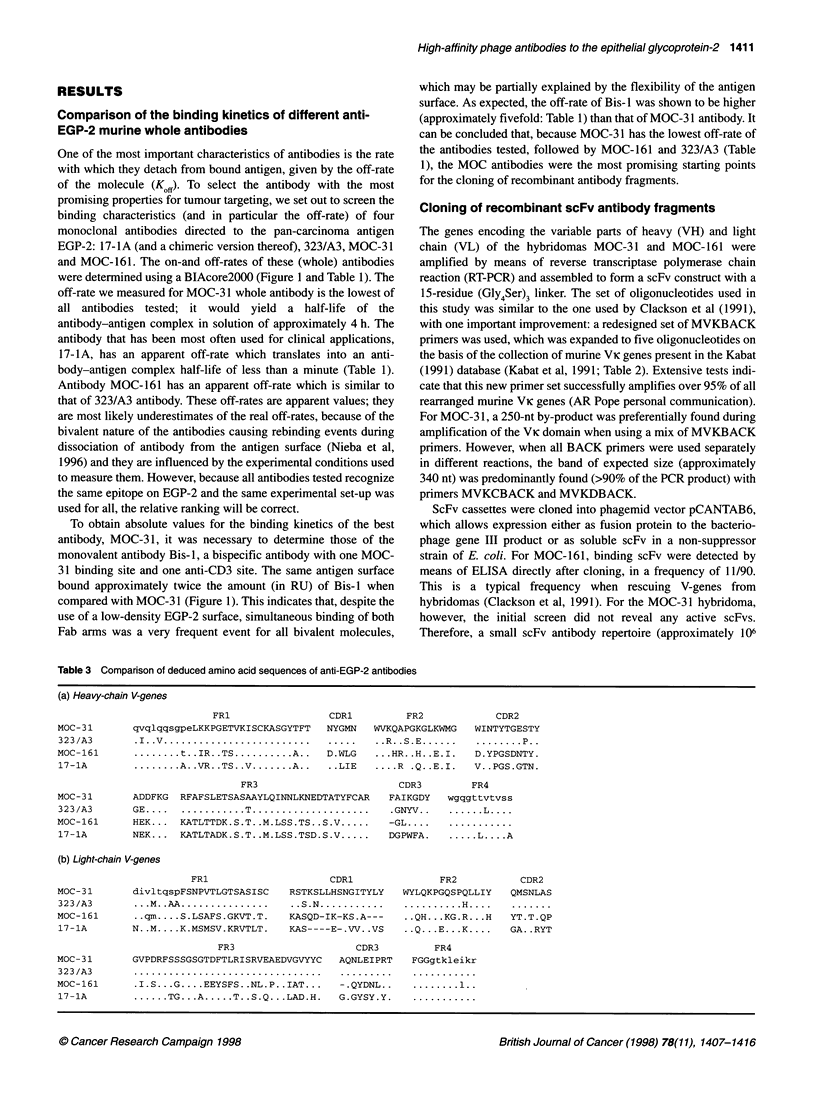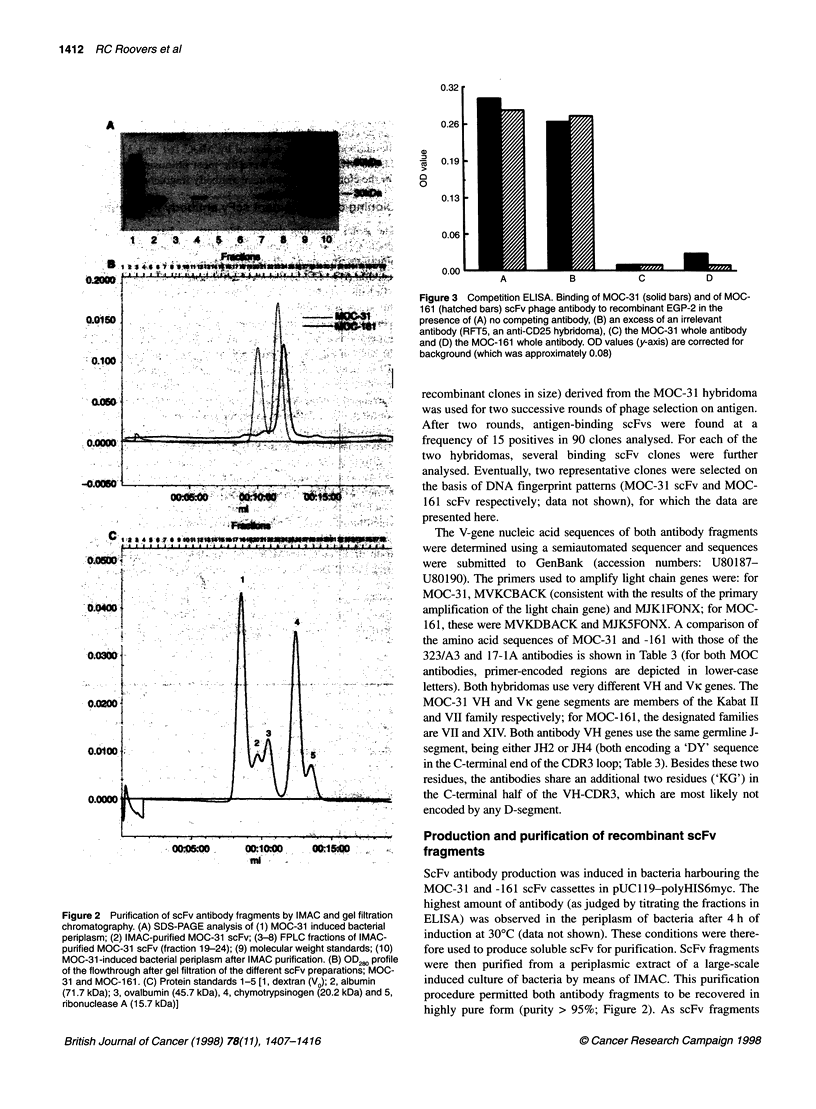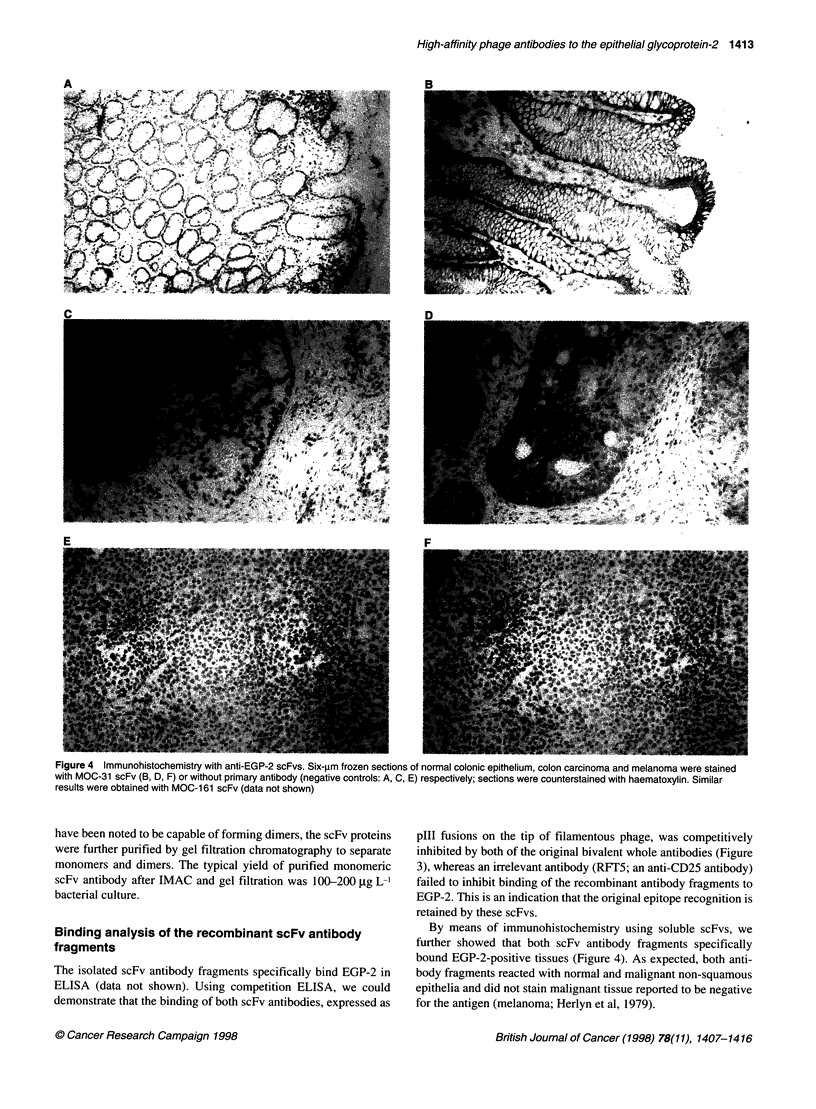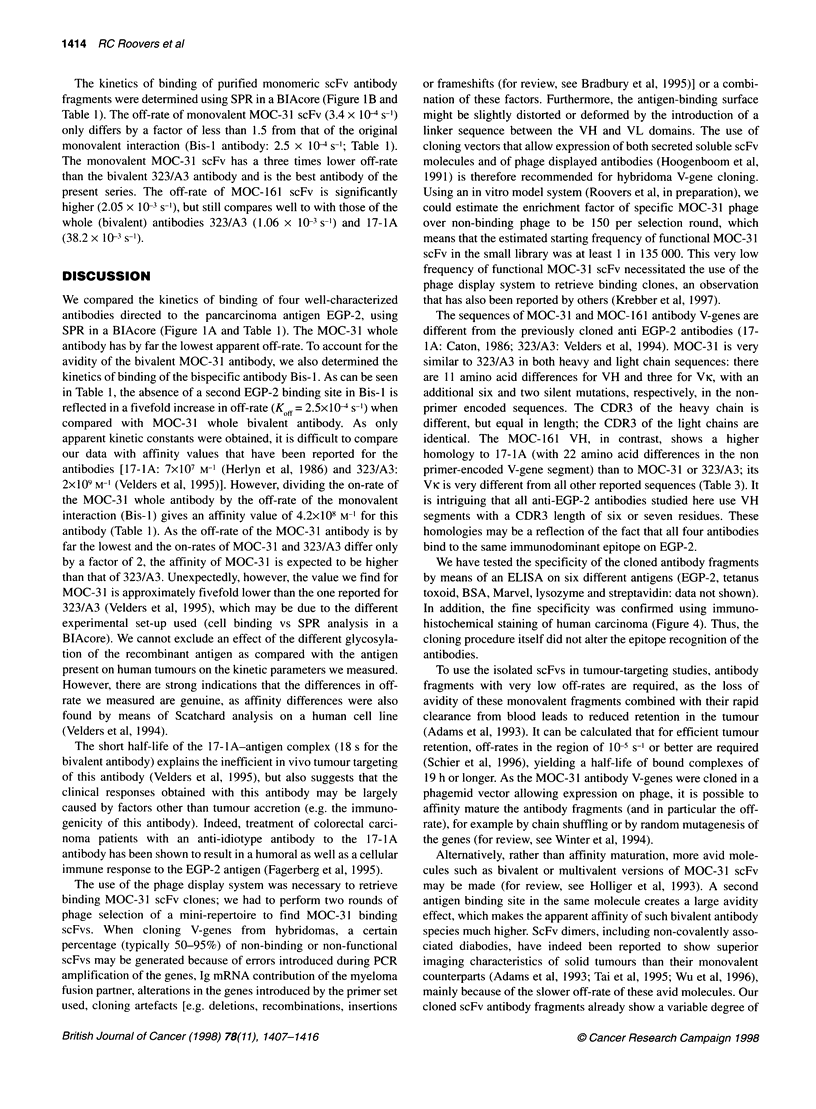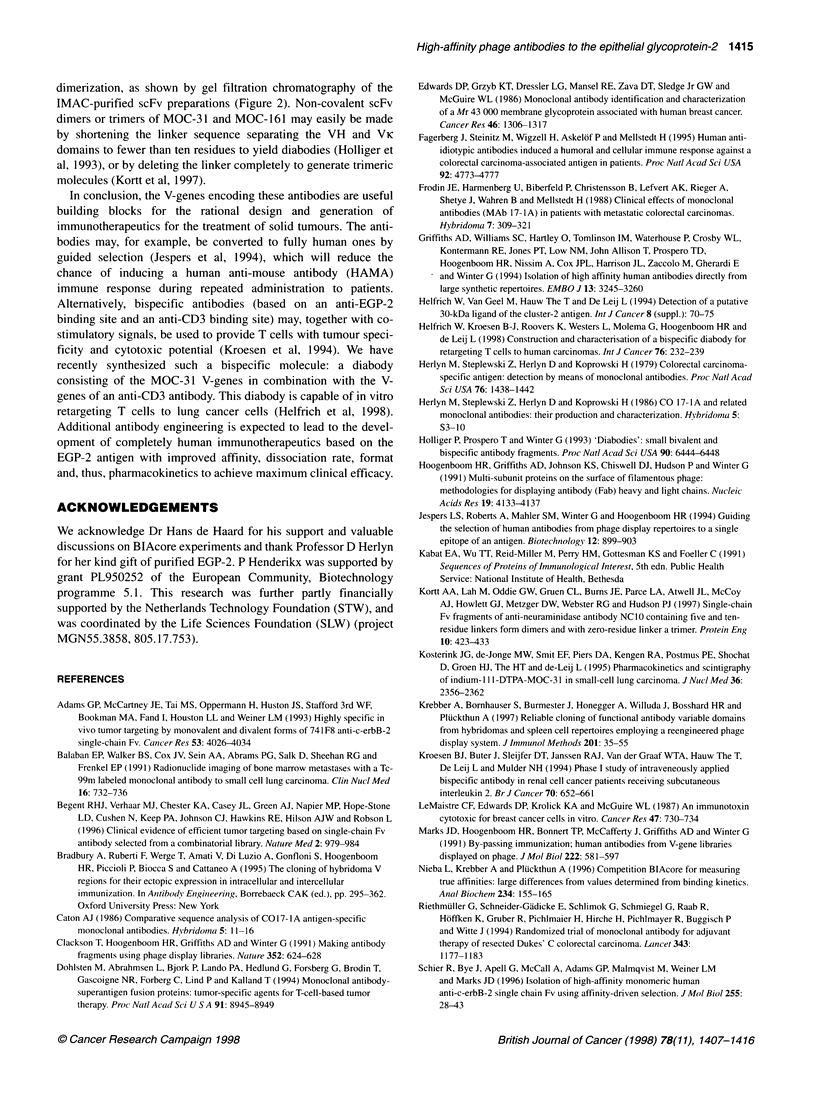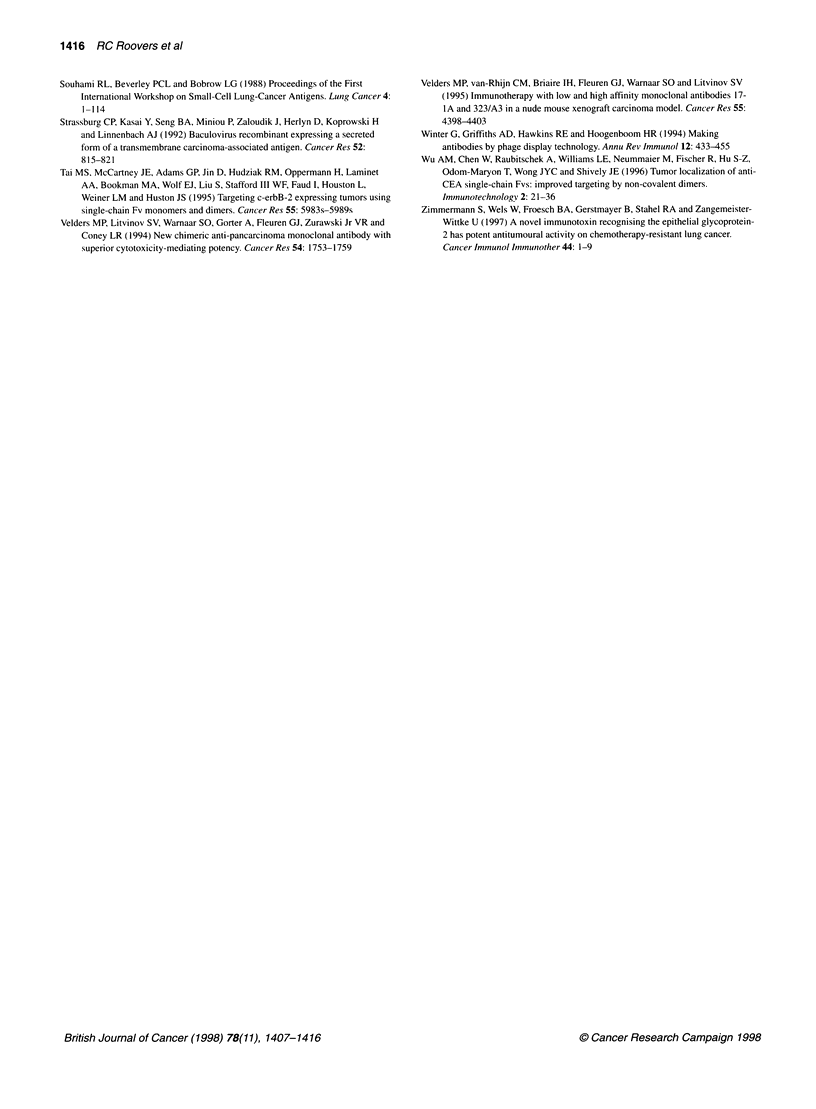# High-affinity recombinant phage antibodies to the pan-carcinoma marker epithelial glycoprotein-2 for tumour targeting.

**DOI:** 10.1038/bjc.1998.700

**Published:** 1998-12

**Authors:** R. C. Roovers, P. Henderikx, W. Helfrich, E. van der Linden, A. Reurs, A. P. de Bruïne, J. W. Arends, L. de Leij, H. R. Hoogenboom

**Affiliations:** Department of Pathology, Maastricht University, The Netherlands.

## Abstract

**Images:**


British Journal of Cancer (1998) 78(11), 1407–1416
© 1998 Cancer Research Campaign

### High-affinity recombinant phage antibodies to the pan-carcinoma marker epithelial glycoprotein-2 for tumour targeting

**RC Roovers[1], P Henderikx[1], W Helfrich[2], E van der Linden[1], A Reurs[1], AP de Bruïne[3], JW Arends[1,3], L de Leij[2] and HR Hoogenboom[2]**

CESAME at Department of Pathology, [1]Maastricht University, PO Box 5800, 6202 AZ Maastricht, The Netherlands; [2]Department of Clinical Immunology, University Hospital Groningen, Oostersingel 59, 9713 EZ Groningen, The Netherlands; [3]University Hospital Maastricht, PO Box 5800, 6202 AZ Maastricht, The Netherlands

**Summary** The tumour-associated antigen epithelial glycoprotein-2 (EGP-2) is a promising target for detection and treatment of a variety of human carcinomas. Antibodies to this antigen have been successfully used in patients for imaging of small-cell lung cancer and for adjuvant treatment of minimal residual disease of colon cancer. We describe here the isolation and complete characterization of high-affinity single-chain variable fragments (scFv) to the EGP-2 antigen. First, the binding kinetics of four murine whole antibodies directed to EGP-2 (17-1A, 323/A3, MOC-31 and MOC-161) were determined using surface plasmon resonance (SPR). The MOC-31 antibody has the lowest apparent off-rate, followed by MOC-161 and 323/A3. The V-genes of the two MOC hybridomas were cloned as scFv in a phage display vector and antigen-binding phage were selected by panning on recombinant antigen. The scFvs compete with the original hybridoma antibodies for binding to antigen and specifically bind to human carcinomas in immunohistochemistry. MOC-31 scFv has an off-rate which is better than those of the bivalent 17-1A and 323/A3 whole antibodies, providing it with an essential characteristic for tumour retention in vivo. The availability of these high-affinity anti-EGP-2 antibody fragments and of their encoding V-genes creates a variety of possibilities for their future use as tumour-targeting vehicles.

**Keywords:** epithelial glycoprotein-2; tumour targeting; single-chain Fv; affinity; phage display

During colorectal carcinogenesis, a number of (membrane) antigens are up-regulated, mutated or differently processed, providing targets for (adjuvant) immunotherapy of the disease. Several tumour antigens have been described and used for targeting or as indicators of progression of disease [e.g. carcinoembryonic antigen (CEA), TAG72, c-erB2, (underglycosylated) MUC-1, p53]. The epithelial glycoprotein-2 (EGP-2, also named CO17-1A antigen, KSA, EGP40 or Ep-CAM) is a tumour-associated antigen present on human simple epithelia and their derived tumours. The abundant expression of EGP-2 on a number of human carcinomas, its limited expression on the luminal side of normal non-squamous epithelia only and the fact that it is not shed into the circulation make this antigen a favourable target for imaging and immunotherapy of cancer. Murine antibodies to EGP-2 have been used in radioimmune detection trials (Balaban et al, 1991; Kosterink et al, 1995), as well as in phase I and II clinical trials (Frodin et al, 1988). The most successful study has been the treatment of minimal residual disease (MRD) in patients with Dukes' C colon carcinoma using the 17-1A antibody (Herlyn et al, 1979). In this study (Riethmüller et al, 1994), an overall 30% reduction in 5-year mortality was observed, proving that passive immunotherapy in an adjuvant setting may be as effective as chemotherapy.

*Received 21 October 1997*
*Revised 19 February 1998*
*Accepted 24 February 1998*

*Correspondence to:* HR Hoogenboom, CESAME at Department of Pathology, University Hospital Maastricht, PO Box 5800, 6202 AZ Maastricht, The Netherlands

Other therapeutic strategies based on the use of antibodies to EGP-2 have also been described. These include the recruitment and activation of T cells by using a fusion of an EGP-2-reactive antibody fragment with the bacterial superantigen staphylococcal enterotoxin A (Dohlsten et al, 1994) or by using bispecific antibodies, directed to both EGP-2 and the T-cell CD3 antigen (Kroesen et al, 1994). In a different approach, the conjugation of anti-EGP-2 antibodies to different bacterial toxins has been shown to yield potent immunotoxins (LeMaistre et al, 1987; Zimmermann et al, 1997). These (pre)clinical studies all underline the possibilities of using antibodies (in different format) to EGP-2 for (adjuvant) immunotherapy.

Careful experimental analysis of scFv variants of an anti-c-erB2 antibody with a range of affinities indicated that there is a clear correlation between affinity increase and enhanced tumour retention (Adams et al, 1993). The availability of high-affinity recombinant anti-EGP-2 antibody fragments would therefore be highly desirable. Indeed, the potential use of such scFv antibody fragments for imaging of human carcinomas has already been demonstrated with a scFv directed to carcinoembryonic antigen (CEA; Begent et al, 1996).

In this study, we first compared the binding kinetics of four murine whole antibodies [17-1A, 323/A3 (Edwards et al, 1986) MOC-31 and MOC-161 (Souhami et al, 1988)] to the antigen EGP-2 using SPR in a BIAcore. Our results show that the MOC-31 antibody has by far the lowest off-rate of all antibodies tested;

MOC-161 has a similar off-rate to the 323/A3 antibody. The V-genes of the two antibodies with lowest off-rate (MOC-31 and MOC-161) were cloned in scFv format and the off-rate and specificity of the scFv antibody fragments determined. These high-affinity anti-EGP-2 scFvs may have important applications in imaging and possibly immunotherapy of different human carcinomas and may provide useful building blocks for further rational therapeutic antibody design.

**Figure 1** Kinetic measurements using SPR in a BIAcore. Sensorgrams showing association and dissociation of different anti-EGP-2 antibodies and antibody fragments: (A) whole antibodies (1, 17–1A; 2, chimeric 17–1A; 3, 323/A3; 4, MOC-31; 5, Bis-1) and (B) antibody fragments (1, MOC-31 scFv; 2, MOC-161 scFv) to the antigen coated to the surface of a sensor chip. Association starts at $t = 130$ s; dissociation starts at $t = 310$ s

### MATERIALS AND METHODS

### Anti-EGP-2 antibodies

The antibodies 17-1A, chimeric 17-1A and 323/A3 were a kind gift from Dr SO Warnaar (Centocor, Leiden, The Netherlands). The two hybridomas cloned in this study [MOC-31 (IgG1/κ) and MOC-161 (IgG2a/κ)] were generated by hybridoma technology (Souhami et al, 1988). Bis-1 bispecific antibody (Kroesen et al, 1994) is produced by a quadroma clone made from the hybridomas MOC-31 (anti EGP-2) and RIV-9 (anti CD3) and was kindly donated by Dr B-J Kroesen (University Hospital Groningen).

### Kinetic measurement using SPR in a BIAcore

Recombinant EGP-2 was expressed in the baculovirus system as described [Strassburg et al, 1992; Helfrich et al, 1994; a kind gift of Professor D Herlyn (the Wistar Institute)]. The antigen was covalently coupled to a CM-5 sensorchip (Pharmacia, Uppsala, Sweden) via free amide chemistry, resulting in a surface of 350 resonance units (RU). All kinetic measurements were performed on this antigen surface. To determine the binding kinetics of different whole antibodies (17-1A, chimeric 17-1A, 323/A3, MOC-161, MOC-31 and Bis-1) and antibody fragments (MOC-31 scFv and MOC-161 scFv), this 'low-density' EGP-2 surface was saturated with antibody (at 200 nM) or antibody fragment using a flow rate of 5 μl $min^{-1}$ (Figure 1). Dissociation rates ($K_{off}$) were then calculated using the BIAevaluation software (Pharmacia) from the sensorgrams depicted in Figure 1. The off-rates were determined by curve fitting on the time interval $t = 315–320$ s, with one exception: because of its very low value, it was impossible to determine the off-rate of the MOC-31 whole antibody in this time period. In this case, an average value of three independent fittings on different parts of the curve is given. As the kinetics of binding of the MOC-161 whole antibody were determined with concentrated hybridoma supernatant, the antibody concentration was unknown and the sensorgram is not shown. However, the same experimental set-up was used, in which the antigen surface was saturated with antibody. All measurements were carried out at room temperature.

**Table 1** Kinetic data and affinities of anti-EGP-2 antibodies and antibody fragments

| Antibody | $K_{on}$ ($10^5$ $M^{-1}$ $s^{-1}$) ± s.e. | $K_{off}$ ($10^{-3}$ $s^{-1}$) ± s.e. (monovalent) | $K_{off}$ ($10^{-3}$ $s^{-1}$) ± s.e. (bivalent) | $K_a$ ($10^8$ $M^{-1}$) | $t_{1/2}$ |
|---|---|---|---|---|---|
| 17-1A | 5.95 ± 0.28 | – | 38.2 ± 1.2[a] | ND | 18 s |
| Chimeric 17-1A | 10.9 ± 0.2 | – | 58.3 ± 2.2[a] | ND | 12 s |
| 323/A3 | 2.51 ± 0.29 | – | 1.06 ± 0.02 | ND | 10 min 54 s |
| Bis-1 | 0.48 ± 0.03 | 0.25 ± 0.03 | – | 1.92 | 46 min 13 s |
| MOC-31 | 1.06 ± 0.16 | – | 0.05 ± 0.01 | 4.24 | 3 h 51 min 03 s |
| MOC-161 | ND | – | 1.55 ± 0.01 | ND | 7 min 27 s |
| MOC-31 scFv | ND | 0.34 ± 0.05 | – | ND | 33 min 59 s |
| MOC-161 scFv | ND | 2.05 ± 0.08 | – | ND | 5 min 38 s |

[a]Because of the very high off-rates of these antibodies, the low-density EGP-2 surface could not be saturated with antibody (Figure 1). These off-rates are therefore understimations of the true values, which may account for the unexpectedly observed difference.

© Cancer Research Campaign 1998

**Table 2** Oligonucleotides used for cloning of murine V-genes

*(a) Primers used for the primary amplification of VH*
MVH1BACK: 5′-AGG T(C/G)(A/C) A(A/G)C TGC AG(C/G) AGT C(A/T)G G-3′
MVH1FOR-2: 5′-TGA GGA GAC GGT GAC CGT GGT CCC TTG GCC CC-3′

*(b) Primers used for the primary amplification of VL*
MVKBACKmix; an equimolar mix of:
-MVKABACK: 5′-GAT GTT TTG ATG ACC CAA ACT CCA-3′
-MVKCBACK: 5′-GAC ATT GTG CT(A/G) ACC CA(A/G) TCT CCA-3′
-MVKDBACK: 5′-GAC ATC CAG ATG AC(T/C/G/A) CAG TCT CCA-3′
-MVKEBACK: 5′-CAA ATT GTT CTC ACC CAG TCT CCA-3′
-MVKFBACK: 5′-GAA AAT GTG CTC ACC CAG TCT CCA-3′
MVKFOR4; an equimolar mix of:
-MJKIFONX; 5′-CCG TTT GAT TTC CAG CTT GGT GCC-3′
-MJK2FONX: 5′-CCG TTT TAT TTC CAG CTT GGT CCC-3′
-MJK4FONX: 5′-CCG TTT TAT TTC CAA CTT TGT CCC-3′
-MJK5FONX: 5′-CCG TTT CAG CTC CAG CTT GGT CCC-3′

*(e) Primers for the synthesis of the linker fragment*
MLINKBACK: 5′-GGG ACC ACG GTC AC C GTC TCC TCA-3′
MLINKFORmix; an equimolar mix of:
-MLINK-A-FOR: 5′-TGG AGT TTG GGT CAT CAA AAC ATC CGA TCC GCC ACC GCC AGA GCC-3′
-MLINK-C-FOR: 5′-TGG AGA CTG GGT (T/C)AG CAC AAT GTC CGA TCC GCC ACC GCC AGA-3′
-MLINK-D-FOR: 5′-TGG AGA CTG NGT CAT CTG GAT GTC CGA TCC GCC ACC GCC AGA GCC-3′
-MLINK-E-FOR: 5′-TGG AGA CTG GGT GAG AAC AAT TTG CGA TCC GCC ACC GCC AGA GCC-3′
-MLINK-F-FOR: 5′-TGG AGA CTG GGT GAG CAC ATT TTC CGA TCC GCC ACC GCC AGA GCC-3′

*(d) Re-amplification primers (to introduce restriction sites in the assembled cassette: sequences encoding restriction sites are underlined)*
MVHIBACKSFI; (introduces a *Sfi*I site at the 3′ end of the assembled scFv cassette):
5′-CAT GCC ATG ACT CGC *GGC CCA GCC GGC C*AT GGC C(C/G)A GGT (C/G)(A/C)A (A/G)CT GCA G(C/G)A GTC (A/T)GG-3′
MVK4FORNOT (introduces a *Not*I site at the 5′ end of the assembled scFv cassette): an equimolar mix of:
-MJK1FORNOT: 5′-GAG TCA TTC TCG ACT TGC GGC CGC CCG TTT GAT TTC CAG CTT GGT GCC-3′
-MJK2FORNOT: 5′-GAG TCA TTC TCG ACT TGC GGC CGC CCG TTT TAT TTC CAG CTT GGT CCC-3′
-MJK4FORNOT: 5′-GAG TCA TTC TCG ACT TGC GGC CGC CCG TTT TAT TTC CAA CTT TGT CCC-3′
-MJK5FORNOT: 5′-GAG TCA TTC TCG ACT TGC GGC CGC CCG TTT CAG CTC CAG CTT GGT CCC-3′

*For sequencing*
pUC-FOR: 5′-CGA CGT TGT AAA ACG ACG GCC AGT-3′
pUC-REVERSE: 5′-CAG GAA ACA GCT ATG AC-3′

Association rates ($K_{on}$) were determined from a plot of [$K_s = (K_{on} \times C + K_{off})$] vs antibody concentration ($C$). Briefly, antibodies were run over the low-density antigen surface at different concentrations and $K_s$ values were determined using the BIAevaluation software. From a plot of the obtained $K_s$ values as function of $C$, $K_{or}$ values were obtained by linear fitting as the slope of the curve. The half-life of the antibody–antigen complex was calculated as $t_{1/2} = \ln 2/K_{off}$ (Table 1).

### Cloning vectors pCANTAB6 is a derivative of pHEN1 (Hoogenboom et al, 1991), carrying an additional stretch of six histidine residues [to allow immobilized metal ion affinity chromatography (IMAC) purification] upstream of the c-*myc*-derived sequence (which allows detection with the 9E10 antibody). ScFv cassettes are cloned as *Sfi*I–*Not*I fragments in frame with the upstream pelB leader sequence and *LacZ* promoter and with the downstream bacteriophage gene III. The vector also contains an amber stop codon between the c-*myc* sequence and gene III, allowing production of soluble scFv in a non-suppressor strain of *Escherichia coli*. Plasmid pUC119–polyHIS6myc (a kind gift from Dr AD Griffiths) is a derivative of pUC119 and carries 5′-*Sfi*I/*Nco*I and 3′-*Not*I cloning sites. ScFv cassettes are cloned in frame with an upstream *LacZ* promoter and *pelB* signal sequence and a downstream cassette of a c-*myc*-derived sequence and six histidine residues. As in the related pUC119–His6mycXba (Griffiths et al, 1994), the bacteriophage gene III is absent.

### *Escherichia coli* strain

TG1: K12, D(*lac-pro*), *supE*, *thi*, *hsdD5*/F′ *traD*36, *proA*$^+$B$^+$, *lacI*$^q$, *lac*ZDM15

### Oligonucleotides

Primers used for the amplification of variable parts of the heavy chain (VH) and of the light chain (VL) of murine immunoglobulin genes, for the synthesis of the linker fragment and for reamplification of the assembled scFv cassettes are listed in Table 2. All primers were purchased from Eurogentec (Liège, Belgium).

### Cloning of immunoglobulin genes

Total cellular RNA was extracted from $10^7$ cells of each of the hybridoma cell lines MOC-31 and MOC-161 by means of the RNazol method (Biotecx Laboratories, Houston, TY, USA). After precipitation, the RNA was dissolved in 20 µl of water and used as template to synthesize cDNA using random hexamer primers (Promega, Madison, WJ, USA) in a 50-µl reverse transcriptase (RT) reaction according to standard procedures. Variable domains of both heavy (VH) and light chain (VL) genes were then amplified

© Cancer Research Campaign 1998 

from cDNA using heavy of light chain-specific primer mixes (see Table 2) and assembled with a linker sequence encoding a 15-residue Gly/Ser sequence by means of Splice Overlap Extension PCR, as described by Clackson et al (1991). The assembled cassette was gel purified, cut with the restriction enzymes *Sfi*I and *Not*I and gel purified again. ScFv cassettes were cloned into *SfiI/Not*I-digested pCANTAB6 DNA and the ligation mix was electroporated into *E. coli* TG1 using standard procedures. Bacteria were plated on 2×TY [1.6% (w/v) trypton, 1% (w/v) yeast extract, 0.5% (w/v) sodium chloride] plates, supplemented with 2% (w/v) glucose and 100 μg $ml^{-1}$ ampicillin, and harvested after overnight growth to form a library of transformants.

### Selection on recombinant, baculovirus-expressed EGP-2 by panning in immunotubes

A small repertoire of transformed bacteria (approximately $10^6$ in size) containing the MOC-31 scFv ligated into pCANTAB6 was rescued with helper phage M13K07 and phage were panned for binding to the antigen EGP-2 [coated at 10 μg $ml^{-1}$ concentration using immunotubes (Maxisorb; Nunc/Life Technologies, Gaithersburg, ND, USA)] as described previously (Marks et al, 1991). Two rounds of selection were performed; after each round, single clones were screened for binding to antigen in enzyme-linked immunosorbent assay (ELISA) as described (see below).

### ELISA and competition ELISA

To identify binding scFvs from the individual clones selected for further analysis, an ELISA using soluble scFv was performed on purified, recombinant EGP-2. Individual bacterial clones were picked and production of soluble scFv was induced by activation of the upstream *LacZ* promoter with isopropyl-β-D-thiogalactopyranoside (IPTG) as described by Marks et al (1991). ELISA plates (Costar, Cambridge, MA, USA) were coated overnight with 1 μg $ml^{-1}$ EGP-2 in phosphate-buffered saline (PBS), washed three times with PBS-T [PBS, 0.5% (v/v) Tween-20], three times with PBS and blocked for 1 h at room temperature (RT) with 2% MPBS [2% (w/v) Marvel – skimmed milk powder – in PBS]. After blocking, induced bacterial supernatants were added [50% (v/v) in 2% MPBS] and incubated for 1.5 h at RT. Bound antibody fragments were detected with the 9E10 antibody [50% (v/v) hybridoma supernatant in 2% MPBS], peroxidase-conjugated rabbit anti-mouse immunoglobulins [Dako, Glostrup, Denmark; 0.1% (v/v) in 2% MPBS] and stained with trimethylbenzidine (TMB) and hydrogen peroxide. Optical density was measured at 450 nm.

For competition ELISA, scFv antibody fragments expressed as pIII fusions on the tip of bacteriophage were detected in the presence of excess whole antibody (because of cross-reactivity of anti-mouse Ig antibodies – used to detect the 9E10 antibody bound to scFvs – with the original whole murine antibodies, this test was not performed with soluble scFvs). Briefly, MOC-31 and MOC-161 phage were rescued with helper phage M13KO7 as described previously (Marks et al, 1991). Approximately $10^{10}$ colony-forming units (cfu) of phage were then mixed with 100 μl of hybridoma supernatant and simultaneously added to different wells of an antigen-coated ELISA plate. Bound phage were detected with a sheep polyclonal antiserum [sheep anti-fd; Pharmacia; 0.02% (v/v) in 2% MPBS], peroxidase-conjugated rabbit anti-goat immunoglobulins [Dako, Glostrup, Denmark; 0.05% (v/v) in 2% MPBS] and stained with TMB/hydrogen peroxide.

### Sequencing

The nucleotide sequences of both the MOC-31 and MOC-161 scFvs were determined using the dideoxy sequencing method of Sanger. Products of the sequencing reaction were analysed on a semiautomated sequencer (Alf Express; Pharmacia). Oligonucleotides used were pUC-FOR and pUC-REV (Table 2).

### Production and purification of soluble scFv

To produce large quantities of both antibody fragments, scFv cassettes were subcloned as *SfiI/Not* I fragments into pUC–119polyHIS6myc, lacking the bacteriophage gene III. This expression plasmid is less toxic to bacteria owing to expression of gene III during induction and thus a higher yield of antibody. Both the supernatant and the periplasmic fraction of bacteria, grown at three different temperatures and harvested after two different time intervals (growth at 20°C, 30°C and 37°C; induction during 4 h or overnight), were first tested for the amount of functional scFv in ELISA.

Five hundred millilitres of 2×TY/A/G [2×TY, supplemented with 100 μg $ml^{-1}$ ampicillin and 2% (w/v) glucose] was inoculated with *E. coli* TG1 harbouring MOC-31 or MOC-161 scFv in pUC119–polyHIS6myc and bacteria were grown at 37°C to an $OD_{600}$ of 1.0. Bacteria were spun down, resuspended in 2×TY containing 100 μg $ml^{-1}$ ampicillin and IPTG to a final concentration of 1 mM and grown for 4 h at 30°C while shaking. After 4 h of induction, bacteria were pelleted and periplasmic fractions were prepared by resuspending the pellet in 8 ml of ice-cold TES (200 mM Tris-HCl, 0.5 mM EDTA, 500 mM sucrose; pH 8.0), adding 12 ml of ice-cold diluted TES (1:3 in water), and incubated on ice for 30 min. Bacteria were spun down (4500 r.p.m., 15 min, 4°C) and the supernatant was collected. The cell pellet was resuspended again in 10 ml of TES, 150 μl of 1 M magnesium sulphate was added and the mix was incubated on ice for 30 min. Cells were spun down again and the supernatant was added to the first periplasmic preparation. EDTA was largely removed by means of dialysis against 20 mM Tris-HCl, 100 mM sodium chloride (pH 8.0). The histidine-tagged scFv fragments were further purified by means of IMAC on Talon resin (Clontech, Palo Alto, CA, USA) using elution with 100 mM imidazole, according to the manufacturer's protocol. Monomeric and dimeric forms of the scFvs were separated by gel filtration chromatography (on a Superdex column; Pharmacia) using a Biologic Apparatus (BioRad, Hercules, USA). Different fractions were finally analysed by means of SDS polyacrylamide gel electrophoresis (SDS-PAGE).

### Immunohistochemical analysis of scFv clones

Six-micron frozen sections (of normal colon epithelium, melanoma and colon carcinoma) were cut, mounted on 3-amino-propyltriethoxy silane (APTS)-coated glass slides, force dried on air and fixed in acetone on ice for 15 min. Slides were force dried on air again, bacterial periplasmic preparations were added [50% (v/v) in PBS/1% bovine serum albumin (BSA)] and incubated for 30 min at 4°C. Slides were washed with PBS and bound scFv antibodies were detected with the 9E10 antibody [50% (v/v) hybridoma supernatant in PBS/1% BSA], peroxidase-conjugated rabbit anti-mouse immunoglobulins [0.1% (v/v) in PBS/1% BSA] and stained with diaminobenzidine (DAB)/hydrogen peroxide. The slides were counterstained with haematoxylin.

© Cancer Research Campaign 1998

### RESULTS

### Comparison of the binding kinetics of different anti-EGP-2 murine whole antibodies

One of the most important characteristics of antibodies is the rate with which they detach from bound antigen, given by the off-rate of the molecule ($K_{off}$). To select the antibody with the most promising properties for tumour targeting, we set out to screen the binding characteristics (and in particular the off-rate) of four monoclonal antibodies directed to the pan-carcinoma antigen EGP-2: 17-1A (and a chimeric version thereof), 323/A3, MOC-31 and MOC-161. The on-and off-rates of these (whole) antibodies were determined using a BIAcore2000 (Figure 1 and Table 1). The off-rate we measured for MOC-31 whole antibody is the lowest of all antibodies tested; it would yield a half-life of the antibody–antigen complex in solution of approximately 4 h. The antibody that has been most often used for clinical applications, 17-1A, has an apparent off-rate which translates into an antibody–antigen complex half-life of less than a minute (Table 1). Antibody MOC-161 has an apparent off-rate which is similar to that of 323/A3 antibody. These off-rates are apparent values; they are most likely underestimates of the real off-rates, because of the bivalent nature of the antibodies causing rebinding events during dissociation of antibody from the antigen surface (Nieba et al, 1996) and they are influenced by the experimental conditions used to measure them. However, because all antibodies tested recognize the same epitope on EGP-2 and the same experimental set-up was used for all, the relative ranking will be correct.

To obtain absolute values for the binding kinetics of the best antibody, MOC-31, it was necessary to determine those of the monovalent antibody Bis-1, a bispecific antibody with one MOC-31 binding site and one anti-CD3 site. The same antigen surface bound approximately twice the amount (in RU) of Bis-1 when compared with MOC-31 (Figure 1). This indicates that, despite the use of a low-density EGP-2 surface, simultaneous binding of both Fab arms was a very frequent event for all bivalent molecules, which may be partially explained by the flexibility of the antigen surface. As expected, the off-rate of Bis-1 was shown to be higher (approximately fivefold: Table 1) than that of MOC-31 antibody. It can be concluded that, because MOC-31 has the lowest off-rate of the antibodies tested, followed by MOC-161 and 323/A3 (Table 1), the MOC antibodies were the most promising starting points for the cloning of recombinant antibody fragments.

### Cloning of recombinant scFv antibody fragments

The genes encoding the variable parts of heavy (VH) and light chain (VL) of the hybridomas MOC-31 and MOC-161 were amplified by means of reverse transcriptase polymerase chain reaction (RT-PCR) and assembled to form a scFv construct with a 15-residue $(Gly_4Ser)_3$ linker. The set of oligonucleotides used in this study was similar to the one used by Clackson et al (1991), with one important improvement: a redesigned set of MVKBACK primers was used, which was expanded to five oligonucleotides on the basis of the collection of murine Vκ genes present in the Kabat (1991) database (Kabat et al, 1991; Table 2). Extensive tests indicate that this new primer set successfully amplifies over 95% of all rearranged murine Vκ genes (AR Pope personal communication). For MOC-31, a 250-nt by-product was preferentially found during amplification of the Vκ domain when using a mix of MVKBACK primers. However, when all BACK primers were used separately in different reactions, the band of expected size (approximately 340 nt) was predominantly found (>90% of the PCR product) with primers MVKCBACK and MVKDBACK.

ScFv cassettes were cloned into phagemid vector pCANTAB6, which allows expression either as fusion protein to the bacteriophage gene III product or as soluble scFv in a non-suppressor strain of *E. coli*. For MOC-161, binding scFv were detected by means of ELISA directly after cloning, in a frequency of 11/90. This is a typical frequency when rescuing V-genes from hybridomas (Clackson et al, 1991). For the MOC-31 hybridoma, however, the initial screen did not reveal any active scFvs. Therefore, a small scFv antibody repertoire (approximately $10^6$

**Table 3** Comparison of deduced amino acid sequences of anti-EGP-2 antibodies

(a) *Heavy-chain V-genes*

| | FR1 | CDR1 | FR2 | CDR2 |
|---|---|---|---|---|
| MOC-31 | qvqlqqsgpeLKKPGETVKISCKASGYTFT | NYGMN | WVKQAPGKGLKWMG | WINTYTGESTY |
| 323/A3 | .I..V......................... | ..... | ..R..S.E...... | ........P.. |
| MOC-161 | ........t..IR..TS..........A.. | D.WLG | ...HR..H..E.I. | D.YPGSDNTY. |
| 17-1A | ........A..VR..TS..V.......A.. | ..LIE | ....R .Q..E.I. | V..PGS.GTN. |

| | FR3 | | CDR3 | FR4 |
|---|---|---|---|---|
| MOC-31 | ADDFKG | RFAFSLETSASAAYLQINNLKNEDTATYFCAR | FAIKGDY | wgqgttvtvss |
| 323/A3 | GE.... | ...........T.................... | .GNYV.. | ......L.... |
| MOC-161 | HEK... | KATLTTDK.S.T..M.LSS.TS..S.V..... | -GL.... | ........... |
| 17-1A | NEK... | KATLTADK.S.T..M.LSS.TSD.S.V..... | DGPWFA. | .....L....A |

(b) *Light-chain V-genes*

| | FR1 | CDR1 | FR2 | CDR2 |
|---|---|---|---|---|
| MOC-31 | divltqspFSNPVTLGTSASISC | RSTKSLLHSNGITYLY | WYLQKPGQSPQLLIY | QMSNLAS |
| 323/A3 | ...M..AA............... | ..S.N........... | ..........H.... | ....... |
| MOC-161 | ..qm....S.LSAFS.GKVT.T. | KASQD-IK-KS.A--- | ..QH...KG.R...H | YT.T.QP |
| 17-1A | N..M....K.MSMSV.KRVTLT. | KAS----E-.VV..VS | ..Q...E...K.... | GA..RYT |

| | FR3 | CDR3 | FR4 |
|---|---|---|---|
| MOC-31 | GVPDRFSSSGSGTDFTLRISRVEAEDVGVYYC | AQNLEIPRT | FGGgtkleikr |
| 323/A3 | ................................ | ......... | ........... |
| MOC-161 | .I.S...G....EEYSFS..NL.P..IAT... | -.QYDNL.. | ........l.. |
| 17-1A | ......TG...A.....T..S.Q...LAD.H. | G.GYSY.Y. | ........... |

*© Cancer Research Campaign 1998* 

**Figure 2** Purification of scFv antibody fragments by IMAC and gel filtration chromatography. (A) SDS-PAGE analysis of (1) MOC-31 induced bacterial periplasm; (2) IMAC-purified MOC-31 scFv; (3–8) FPLC fractions of IMAC-purified MOC-31 scFv (fraction 19–24); (9) molecular weight standards; (10) MOC-31-induced bacterial periplasm after IMAC purification. (B) $OD_{280}$ profile of the flowthrough after gel filtration of the different scFv preparations; MOC-31 and MOC-161. (C) Protein standards 1–5 [1, dextran ($V_0$); 2, albumin (71.7 kDa); 3, ovalbumin (45.7 kDa), 4, chymotrypsinogen (20.2 kDa) and 5, ribonuclease A (15.7 kDa)]

**Figure 3** Competition ELISA. Binding of MOC-31 (solid bars) and of MOC-161 (hatched bars) scFv phage antibody to recombinant EGP-2 in the presence of (A) no competing antibody, (B) an excess of an irrelevant antibody (RFT5, an anti-CD25 hybridoma), (C) the MOC-31 whole antibody and (D) the MOC-161 whole antibody. OD values (*y*-axis) are corrected for background (which was approximately 0.08)

recombinant clones in size) derived from the MOC-31 hybridoma was used for two successive rounds of phage selection on antigen. After two rounds, antigen-binding scFvs were found at a frequency of 15 positives in 90 clones analysed. For each of the two hybridomas, several binding scFv clones were further analysed. Eventually, two representative clones were selected on the basis of DNA fingerprint patterns (MOC-31 scFv and MOC-161 scFv respectively; data not shown), for which the data are presented here.

The V-gene nucleic acid sequences of both antibody fragments were determined using a semiautomated sequencer and sequences were submitted to GenBank (accession numbers: U80187–U80190). The primers used to amplify light chain genes were: for MOC-31, MVKCBACK (consistent with the results of the primary amplification of the light chain gene) and MJK1FONX; for MOC-161, these were MVKDBACK and MJK5FONX. A comparison of the amino acid sequences of MOC-31 and -161 with those of the 323/A3 and 17-1A antibodies is shown in Table 3 (for both MOC antibodies, primer-encoded regions are depicted in lower-case letters). Both hybridomas use very different VH and Vκ genes. The MOC-31 VH and Vκ gene segments are members of the Kabat II and VII family respectively; for MOC-161, the designated families are VII and XIV. Both antibody VH genes use the same germline J-segment, being either JH2 or JH4 (both encoding a 'DY' sequence in the C-terminal end of the CDR3 loop; Table 3). Besides these two residues, the antibodies share an additional two residues ('KG') in the C-terminal half of the VH-CDR3, which are most likely not encoded by any D-segment.

### Production and purification of recombinant scFv fragments

ScFv antibody production was induced in bacteria harbouring the MOC-31 and -161 scFv cassettes in pUC119–polyHIS6myc. The highest amount of antibody (as judged by titrating the fractions in ELISA) was observed in the periplasm of bacteria after 4 h of induction at 30°C (data not shown). These conditions were therefore used to produce soluble scFv for purification. ScFv fragments were then purified from a periplasmic extract of a large-scale induced culture of bacteria by means of IMAC. This purification procedure permitted both antibody fragments to be recovered in highly pure form (purity > 95%; Figure 2). As scFv fragments

*© Cancer Research Campaign 1998*

**Figure 4** Immunohistochemistry with anti-EGP-2 scFvs. Six-µm frozen sections of normal colonic epithelium, colon carcinoma and melanoma were stained with MOC-31 scFv (B, D, F) or without primary antibody (negative controls: A, C, E) respectively; sections were counterstained with haematoxylin. Similar results were obtained with MOC-161 scFv (data not shown)

have been noted to be capable of forming dimers, the scFv proteins were further purified by gel filtration chromatography to separate monomers and dimers. The typical yield of purified monomeric scFv antibody after IMAC and gel filtration was 100–200 µg $L^{-1}$ bacterial culture.

### Binding analysis of the recombinant scFv antibody fragments

The isolated scFv antibody fragments specifically bind EGP-2 in ELISA (data not shown). Using competition ELISA, we could demonstrate that the binding of both scFv antibodies, expressed as pIII fusions on the tip of filamentous phage, was competitively inhibited by both of the original bivalent whole antibodies (Figure 3), whereas an irrelevant antibody (RFT5; an anti-CD25 antibody) failed to inhibit binding of the recombinant antibody fragments to EGP-2. This is an indication that the original epitope recognition is retained by these scFvs.

By means of immunohistochemistry using soluble scFvs, we further showed that both scFv antibody fragments specifically bound EGP-2-positive tissues (Figure 4). As expected, both antibody fragments reacted with normal and malignant non-squamous epithelia and did not stain malignant tissue reported to be negative for the antigen (melanoma; Herlyn et al, 1979).

© Cancer Research Campaign 1998

The kinetics of binding of purified monomeric scFv antibody fragments were determined using SPR in a BIAcore (Figure 1B and Table 1). The off-rate of monovalent MOC-31 scFv ($3.4 \times 10^{-4}$ $s^{-1}$) only differs by a factor of less than 1.5 from that of the original monovalent interaction (Bis-1 antibody: $2.5 \times 10^{-4}$ $s^{-1}$; Table 1). The monovalent MOC-31 scFv has a three times lower off-rate than the bivalent 323/A3 antibody and is the best antibody of the present series. The off-rate of MOC-161 scFv is significantly higher ($2.05 \times 10^{-3}$ $s^{-1}$), but still compares well to with those of the whole (bivalent) antibodies 323/A3 ($1.06 \times 10^{-3}$ $s^{-1}$) and 17-1A ($38.2 \times 10^{-3}$ $s^{-1}$).

### DISCUSSION

We compared the kinetics of binding of four well-characterized antibodies directed to the pancarcinoma antigen EGP-2, using SPR in a BIAcore (Figure 1A and Table 1). The MOC-31 whole antibody has by far the lowest apparent off-rate. To account for the avidity of the bivalent MOC-31 antibody, we also determined the kinetics of binding of the bispecific antibody Bis-1. As can be seen in Table 1, the absence of a second EGP-2 binding site in Bis-1 is reflected in a fivefold increase in off-rate ($K_{off} = 2.5 \times 10^{-4}$ $s^{-1}$) when compared with MOC-31 whole bivalent antibody. As only apparent kinetic constants were obtained, it is difficult to compare our data with affinity values that have been reported for the antibodies [17-1A: $7 \times 10^{7}$ $M^{-1}$ (Herlyn et al, 1986) and 323/A3: $2 \times 10^{9}$ $M^{-1}$ (Velders et al, 1995)]. However, dividing the on-rate of the MOC-31 whole antibody by the off-rate of the monovalent interaction (Bis-1) gives an affinity value of $4.2 \times 10^{8}$ $M^{-1}$ for this antibody (Table 1). As the off-rate of the MOC-31 antibody is by far the lowest and the on-rates of MOC-31 and 323/A3 differ only by a factor of 2, the affinity of MOC-31 is expected to be higher than that of 323/A3. Unexpectedly, however, the value we find for MOC-31 is approximately fivefold lower than the one reported for 323/A3 (Velders et al, 1995), which may be due to the different experimental set-up used (cell binding vs SPR analysis in a BIAcore). We cannot exclude an effect of the different glycosylation of the recombinant antigen as compared with the antigen present on human tumours on the kinetic parameters we measured. However, there are strong indications that the differences in off-rate we measured are genuine, as affinity differences were also found by means of Scatchard analysis on a human cell line (Velders et al, 1994).

The short half-life of the 17-1A–antigen complex (18 s for the bivalent antibody) explains the inefficient in vivo tumour targeting of this antibody (Velders et al, 1995), but also suggests that the clinical responses obtained with this antibody may be largely caused by factors other than tumour accretion (e.g. the immunogenicity of this antibody). Indeed, treatment of colorectal carcinoma patients with an anti-idiotype antibody to the 17-1A antibody has been shown to result in a humoral as well as a cellular immune response to the EGP-2 antigen (Fagerberg et al, 1995).

The use of the phage display system was necessary to retrieve binding MOC-31 scFv clones; we had to perform two rounds of phage selection of a mini-repertoire to find MOC-31 binding scFvs. When cloning V-genes from hybridomas, a certain percentage (typically 50–95%) of non-binding or non-functional scFvs may be generated because of errors introduced during PCR amplification of the genes, Ig mRNA contribution of the myeloma fusion partner, alterations in the genes introduced by the primer set used, cloning artefacts [e.g. deletions, recombinations, insertions or frameshifts (for review, see Bradbury et al, 1995)] or a combination of these factors. Furthermore, the antigen-binding surface might be slightly distorted or deformed by the introduction of a linker sequence between the VH and VL domains. The use of cloning vectors that allow expression of both secreted soluble scFv molecules and of phage displayed antibodies (Hoogenboom et al, 1991) is therefore recommended for hybridoma V-gene cloning. Using an in vitro model system (Roovers et al, in preparation), we could estimate the enrichment factor of specific MOC-31 phage over non-binding phage to be 150 per selection round, which means that the estimated starting frequency of functional MOC-31 scFv in the small library was at least 1 in 135 000. This very low frequency of functional MOC-31 scFv necessitated the use of the phage display system to retrieve binding clones, an observation that has also been reported by others (Krebber et al, 1997).

The sequences of MOC-31 and MOC-161 antibody V-genes are different from the previously cloned anti EGP-2 antibodies (17-1A: Caton, 1986; 323/A3: Velders et al, 1994). MOC-31 is very similar to 323/A3 in both heavy and light chain sequences: there are 11 amino acid differences for VH and three for Vκ, with an additional six and two silent mutations, respectively, in the non-primer encoded sequences. The CDR3 of the heavy chain is different, but equal in length; the CDR3 of the light chains are identical. The MOC-161 VH, in contrast, shows a higher homology to 17-1A (with 22 amino acid differences in the non primer-encoded V-gene segment) than to MOC-31 or 323/A3; its Vκ is very different from all other reported sequences (Table 3). It is intriguing that all anti-EGP-2 antibodies studied here use VH segments with a CDR3 length of six or seven residues. These homologies may be a reflection of the fact that all four antibodies bind to the same immunodominant epitope on EGP-2.

We have tested the specificity of the cloned antibody fragments by means of an ELISA on six different antigens (EGP-2, tetanus toxoid, BSA, Marvel, lysozyme and streptavidin: data not shown). In addition, the fine specificity was confirmed using immunohistochemical staining of human carcinoma (Figure 4). Thus, the cloning procedure itself did not alter the epitope recognition of the antibodies.

To use the isolated scFvs in tumour-targeting studies, antibody fragments with very low off-rates are required, as the loss of avidity of these monovalent fragments combined with their rapid clearance from blood leads to reduced retention in the tumour (Adams et al, 1993). It can be calculated that for efficient tumour retention, off-rates in the region of $10^{-5}$ $s^{-1}$ or better are required (Schier et al, 1996), yielding a half-life of bound complexes of 19 h or longer. As the MOC-31 antibody V-genes were cloned in a phagemid vector allowing expression on phage, it is possible to affinity mature the antibody fragments (and in particular the off-rate), for example by chain shuffling or by random mutagenesis of the genes (for review, see Winter et al, 1994).

Alternatively, rather than affinity maturation, more avid molecules such as bivalent or multivalent versions of MOC-31 scFv may be made (for review, see Holliger et al, 1993). A second antigen binding site in the same molecule creates a large avidity effect, which makes the apparent affinity of such bivalent antibody species much higher. ScFv dimers, including non-covalently associated diabodies, have indeed been reported to show superior imaging characteristics of solid tumours than their monovalent counterparts (Adams et al, 1993; Tai et al, 1995; Wu et al, 1996), mainly because of the slower off-rate of these avid molecules. Our cloned scFv antibody fragments already show a variable degree of

© Cancer Research Campaign 1998

dimerization, as shown by gel filtration chromatography of the IMAC-purified scFv preparations (Figure 2). Non-covalent scFv dimers or trimers of MOC-31 and MOC-161 may easily be made by shortening the linker sequence separating the VH and Vκ domains to fewer than ten residues to yield diabodies (Holliger et al, 1993), or by deleting the linker completely to generate trimeric molecules (Kortt et al, 1997).

In conclusion, the V-genes encoding these antibodies are useful building blocks for the rational design and generation of immunotherapeutics for the treatment of solid tumours. The antibodies may, for example, be converted to fully human ones by guided selection (Jespers et al, 1994), which will reduce the chance of inducing a human anti-mouse antibody (HAMA) immune response during repeated administration to patients. Alternatively, bispecific antibodies (based on an anti-EGP-2 binding site and an anti-CD3 binding site) may, together with co-stimulatory signals, be used to provide T cells with tumour specificity and cytotoxic potential (Kroesen et al, 1994). We have recently synthesized such a bispecific molecule: a diabody consisting of the MOC-31 V-genes in combination with the V-genes of an anti-CD3 antibody. This diabody is capable of in vitro retargeting T cells to lung cancer cells (Helfrich et al, 1998). Additional antibody engineering is expected to lead to the development of completely human immunotherapeutics based on the EGP-2 antigen with improved affinity, dissociation rate, format and, thus, pharmacokinetics to achieve maximum clinical efficacy.

### ACKNOWLEDGEMENTS

We acknowledge Dr Hans de Haard for his support and valuable discussions on BIAcore experiments and thank Professor D Herlyn for her kind gift of purified EGP-2. P Henderikx was supported by grant PL950252 of the European Community, Biotechnology programme 5.1. This research was further partly financially supported by the Netherlands Technology Foundation (STW), and was coordinated by the Life Sciences Foundation (SLW) (project MGN55.3858, 805.17.753).

### REFERENCES

Adams GP, McCartney JE, Tai MS, Oppermann H, Huston JS, Stafford 3rd WF, Bookman MA, Fand I, Houston LL and Weiner LM (1993) Highly specific in vivo tumor targeting by monovalent and divalent forms of 741F8 anti-c-erbB-2 single-chain Fv. *Cancer Res* **53**: 4026–4034

Balaban EP, Walker BS, Cox JV, Sein AA, Abrams PG, Salk D, Sheehan RG and Frenkel EP (1991) Radionuclide imaging of bone marrow metastases with a Tc-99m labeled monoclonal antibody to small cell lung carcinoma. *Clin Nucl Med* **16**: 732–736

Begent RHJ, Verhaar MJ, Chester KA, Casey JL, Green AJ, Napier MP, Hope-Stone LD, Cushen N, Keep PA, Johnson CJ, Hawkins RE, Hilson AJW and Robson L (1996) Clinical evidence of efficient tumor targeting based on single-chain Fv antibody selected from a combinatorial library. *Nature Med* **2**: 979–984

Bradbury A, Ruberti F, Werge T, Amati V, Di Luzio A, Gonfloni S, Hoogenboom HR, Piccioli P, Biocca S and Cattaneo A (1995) The cloning of hybridoma V regions for their ectopic expression in intracellular and intercellular immunization. In *Antibody Engineering*, Borrebaeck CAK (ed.), pp. 295–362. Oxford University Press: New York

Caton AJ (1986) Comparative sequence analysis of CO17-1A antigen-specific monoclonal antibodies. *Hybridoma* **5**: 11–16

Clackson T, Hoogenboom HR, Griffiths AD and Winter G (1991) Making antibody fragments using phage display libraries. *Nature* **352**: 624–628

Dohlsten M, Abrahmsen L, Bjork P, Lando PA, Hedlund G, Forsberg G, Brodin T, Gascoigne NR, Forberg C, Lind P and Kalland T (1994) Monoclonal antibody-superantigen fusion proteins: tumor-specific agents for T-cell-based tumor therapy. *Proc Natl Acad Sci U S A* **91**: 8945–8949

Edwards DP, Grzyb KT, Dressler LG, Mansel RE, Zava DT, Sledge Jr GW and McGuire WL (1986) Monoclonal antibody identification and characterization of a *Mr* 43 000 membrane glycoprotein associated with human breast cancer. *Cancer Res* **46**: 1306–1317

Fagerberg J, Steinitz M, Wigzell H, Askelöf P and Mellstedt H (1995) Human anti-idiotypic antibodies induced a humoral and cellular immune response against a colorectal carcinoma-associated antigen in patients. *Proc Natl Acad Sci USA* **92**: 4773–4777

Frodin JE, Harmenberg U, Biberfeld P, Christensson B, Lefvert AK, Rieger A, Shetye J, Wahren B and Mellstedt H (1988) Clinical effects of monoclonal antibodies (MAb 17-1A) in patients with metastatic colorectal carcinomas. *Hybridoma* **7**: 309–321

Griffiths AD, Williams SC, Hartley O, Tomlinson IM, Waterhouse P, Crosby WL, Kontermann RE, Jones PT, Low NM, John Allison T, Prospero TD, Hoogenboom HR, Nissim A, Cox JPL, Harrison JL, Zaccolo M, Gherardi E and Winter G (1994) Isolation of high affinity human antibodies directly from large synthetic repertoires. *EMBO J* **13**: 3245–3260

Helfrich W, Van Geel M, Hauw The T and De Leij L (1994) Detection of a putative 30-kDa ligand of the cluster-2 antigen. *Int J Cancer* **8** (suppl.): 70–75

Helfrich W, Kroesen B-J, Roovers K, Westers L, Molema G, Hoogenboom HR and de Leij L (1998) Construction and characterisation of a bispecific diabody for retargeting T cells to human carcinomas. *Int J Cancer* **76**: 232–239

Herlyn M, Steplewski Z, Herlyn D and Koprowski H (1979) Colorectal carcinoma-specific antigen: detection by means of monoclonal antibodies. *Proc Natl Acad Sci USA* **76**: 1438–1442

Herlyn M, Steplewski Z, Herlyn D and Koprowski H (1986) CO 17-1A and related monoclonal antibodies: their production and characterization. *Hybridoma* **5**: S3–10

Holliger P, Prospero T and Winter G (1993) 'Diabodies': small bivalent and bispecific antibody fragments. *Proc Natl Acad Sci USA* **90**: 6444–6448

Hoogenboom HR, Griffiths AD, Johnson KS, Chiswell DJ, Hudson P and Winter G (1991) Multi-subunit proteins on the surface of filamentous phage: methodologies for displaying antibody (Fab) heavy and light chains. *Nucleic Acids Res* **19**: 4133–4137

Jespers LS, Roberts A, Mahler SM, Winter G and Hoogenboom HR (1994) Guiding the selection of human antibodies from phage display repertoires to a single epitope of an antigen. *Biotechnology* **12**: 899–903

Kabat EA, Wu TT, Reid-Miller M, Perry HM, Gottesman KS and Foeller C (1991) *Sequences of Proteins of Immunological Interest*, 5th edn. Public Health Service: National Institute of Health, Bethesda

Kortt AA, Lah M, Oddie GW, Gruen CL, Burns JE, Parce LA, Atwell JL, McCoy AJ, Howlett GJ, Metzger DW, Webster RG and Hudson PJ (1997) Single-chain Fv fragments of anti-neuraminidase antibody NC10 containing five and ten-residue linkers form dimers and with zero-residue linker a trimer. *Protein Eng* **10**: 423–433

Kosterink JG, de-Jonge MW, Smit EF, Piers DA, Kengen RA, Postmus PE, Shochat D, Groen HJ, The HT and de-Leij L (1995) Pharmacokinetics and scintigraphy of indium-111-DTPA-MOC-31 in small-cell lung carcinoma. *J Nucl Med* **36**: 2356–2362

Krebber A, Bornhauser S, Burmester J, Honegger A, Willuda J, Bosshard HR and Plückthun A (1997) Reliable cloning of functional antibody variable domains from hybridomas and spleen cell repertoires employing a reengineered phage display system. *J Immunol Methods* **201**: 35–55

Kroesen BJ, Buter J, Sleijfer DT, Janssen RAJ, Van der Graaf WTA, Hauw The T, De Leij L and Mulder NH (1994) Phase I study of intraveneously applied bispecific antibody in renal cell cancer patients receiving subcutaneous interleukin 2. *Br J Cancer* **70**: 652–661

LeMaistre CF, Edwards DP, Krolick KA and McGuire WL (1987) An immunotoxin cytotoxic for breast cancer cells in vitro. *Cancer Res* **47**: 730–734

Marks JD, Hoogenboom HR, Bonnert TP, McCafferty J, Griffiths AD and Winter G (1991) By-passing immunization; human antibodies from V-gene libraries displayed on phage. *J Mol Biol* **222**: 581–597

Nieba L, Krebber A and Plückthun A (1996) Competition BIAcore for measuring true affinities: large differences from values determined from binding kinetics. *Anal Biochem* **234**: 155–165

Riethmüller G, Schneider-Gädicke E, Schlimok G, Schmiegel G, Raab R, Höffken K, Gruber R, Pichlmaier H, Hirche H, Pichlmayer R, Buggisch P and Witte J (1994) Randomized trial of monoclonal antibody for adjuvant therapy of resected Dukes' C colorectal carcinoma. *Lancet* **343**: 1177–1183

Schier R, Bye J, Apell G, McCall A, Adams GP, Malmqvist M, Weiner LM and Marks JD (1996) Isolation of high-affinity monomeric human anti-c-erbB-2 single chain Fv using affinity-driven selection. *J Mol Biol* **255**: 28–43

© Cancer Research Campaign 1998 

Souhami RL, Beverley PCL and Bobrow LG (1988) Proceedings of the First International Workshop on Small-Cell Lung-Cancer Antigens. *Lung Cancer* **4**: 1–114

Strassburg CP, Kasai Y, Seng BA, Miniou P, Zaloudik J, Herlyn D, Koprowski H and Linnenbach AJ (1992) Baculovirus recombinant expressing a secreted form of a transmembrane carcinoma-associated antigen. *Cancer Res* **52**: 815–821

Tai MS, McCartney JE, Adams GP, Jin D, Hudziak RM, Oppermann H, Laminet AA, Bookman MA, Wolf EJ, Liu S, Stafford III WF, Faud I, Houston L, Weiner LM and Huston JS (1995) Targeting c-erbB-2 expressing tumors using single-chain Fv monomers and dimers. *Cancer Res* **55**: 5983s–5989s

Velders MP, Litvinov SV, Warnaar SO, Gorter A, Fleuren GJ, Zurawski Jr VR and Coney LR (1994) New chimeric anti-pancarcinoma monoclonal antibody with superior cytotoxicity-mediating potency. *Cancer Res* **54**: 1753–1759

Velders MP, van-Rhijn CM, Briaire IH, Fleuren GJ, Warnaar SO and Litvinov SV (1995) Immunotherapy with low and high affinity monoclonal antibodies 17-1A and 323/A3 in a nude mouse xenograft carcinoma model. *Cancer Res* **55**: 4398–4403

Winter G, Griffiths AD, Hawkins RE and Hoogenboom HR (1994) Making antibodies by phage display technology. *Annu Rev Immunol* **12**: 433–455

Wu AM, Chen W, Raubitschek A, Williams LE, Neummaier M, Fischer R, Hu S-Z, Odom-Maryon T, Wong JYC and Shively JE (1996) Tumor localization of anti-CEA single-chain Fvs: improved targeting by non-covalent dimers. *Immunotechnology* **2**: 21–36

Zimmermann S, Wels W, Froesch BA, Gerstmayer B, Stahel RA and Zangemeister-Wittke U (1997) A novel immunotoxin recognising the epithelial glycoprotein-2 has potent antitumoural activity on chemotherapy-resistant lung cancer. *Cancer Immunol Immunother* **44**: 1–9

© Cancer Research Campaign 1998